# Nitrogen-doped Carbon Microfiber with Wrinkled Surface for High Performance Supercapacitors

**DOI:** 10.1038/srep21750

**Published:** 2016-02-18

**Authors:** Ruili Liu, Lixia Pan, Jianzhong Jiang, Xin Xi, Xiaoxue Liu, Dongqing Wu

**Affiliations:** 1 State Key Laboratory of Advanced Optical Communication Systems and Networks, National Engineering Lab for TFT-LCD Materials and Technologies, Department of Electronic Engineering, Shanghai Jiao Tong University, Shanghai 200240, China; 2 Department of Chemical Engineering, School of Environment and Chemical Engineering, Shanghai University, Shanghai 200444, China; 3 School of Chemistry and Chemical Engineering, Shanghai Jiao Tong University, Shanghai 200240, China

## Abstract

In this work, nitrogen-doped carbon microfiber (NCMF) is fabricated via a facile co-assembly of natural silk and graphene oxide (GO) and the following thermal treatment. The amphiphilic nature of GO endows NCMF a crumpled surface with a high surface area of 115 m^2^ g^−1^. As the binder-free electrode in electrical double-layer capacitors, NCMF shows an excellent capacitance of 196 F g^−1^ at scan rate of 5 mV s^−1^, which is almost four times higher than that of the pristine CMF from silk (55 F g^−1^). Additionally, the capacitance of NCMF can be kept around 92 F g^−1^ at a high scan rate of 300 mV s^−1^ even after 10000 cycles. More importantly, a high energy density (≈22.7 μW h cm^**−**2^) and power density (≈10.26 mW cm^**−**2^) are achieved by the all-solid-state supercapacitor based on NCMF.

The high power density, good operational safety, and long cycling life of electrical double-layer capacitors (EDLCs, also known as supercapacitors) make them highly desirable energy storage devices to fill the gap between batteries and traditional capacitors^1^,^2^,^3^. However, the relatively low energy density of EDLCs is still the major obstacle retarding their practical applications^4^,^5^. Generally, the electrochemical behaviors of EDLCs including capacitances and cycling stability highly depend on the compositions and structures of their electrodes^6^,^7^,^8^. With this respect, the delicate design and synthesis of unprecedented electrode materials is an urgent task for the development of high performance EDLCs.

Carbon microfibers (CMF) with the diameter of 5–10 μm are very appealing electrode materials for EDLCs since their linear structure with high aspect ratio provides one-dimensional (1D) pathway for the rapid transportation of electrons and they can be directly used as the active films without any binders. Generally, CMFs are fabricated from pitch, phenolic resins, polyaniline, polyacrylonitrile and so on. Because the energy storage mechanism of EDLCs is based on the reversible ion adsorption on the electrolyte/electrode interfaces, the improvement on the accessible surface area of CMF is thus crucial for the elevation of their electrochemical performances in EDLCs^3^. For this purpose, an activation process is usually required to generate porous architecture in CMFs, which inevitably increases the cost and manufacturing difficulty of them. On the other hand, it has been found that the doping of carbon with heteroatoms such as nitrogen (N) or sulfur (S) can effectively improve the electronic conductivity and surface wettability of the carbon based electrodes^6^,^7^,^9^. Moreover, the pseudo-capacitance contributed by the nitrogen containing groups is also desirable for the performance promotion of the EDLC electrodes^9^,^10^,^11^.

Herein, we report a facile method to prepare nitrogen-doped CMF (NCMF) via the co-assembly of natural silk and graphene oxide (GO) in aqueous solution and the following thermal treatment. As a two-dimensional amphiphilic polymer, GO can form a wrinkled shell around the silk derived carbon fibre. With the highly crumpled surface, NCMF exhibits a surface area as 115 m^2^ g^−1^, which is much higher than the silk-derived pristine CMF with almost undetectable surface area. Serving as the binder-free electrode in EDLCs, NCMF shows an excellent capacitance of 196 F g^−1^ at scan rate of 5 mV s^−1^, which is superior to those of the pristine CMF (55 F g^−1^) and the mechanically mixed CMF and graphene (CMF&G, 111 F g^−1^). The capacitance of NCMF is retained as 92 F g^−1^ even at a high scan rate of 300 mV s^−1^ after 10000 cycles. More importantly, the all-solid-state supercapacitor (ASSS) based on NCMF also exhibits high specific capacitance (255 mF cm^−2^ at 2 mV s^**−**1^) and excellent rate capability (85.5 mF cm^−2^ at 300 mV s^**−**1^), leading to a high energy density (≈22.7 μW h cm^**−**2^) and power density (≈10.26 mW cm^**−**2^).

## Results and Discussion

**Figure 1:**
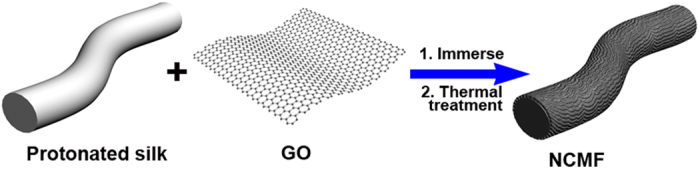
Schematic illustration of the synthesis procedures for NCMF.

The synthesis procedures of NCMF are illustrated in Fig. 1. A piece of silk sheet was first treated with diluted HCl to allow the protonation of the nitrogen containing amide groups on silk. Subsequently, the protonated silk was immersed in the suspension of GO. During this process, the ionic interactions between negatively charged GO and positively charged silk enable GO nanosheets to wrap around silk, which thus leads to the formation of the core/shell structured composites (Fig. S1a). The following thermal treatment of the resulting composites in nitrogen flow can both convert silk to carbon microfibers and reduce the GO shell at the same time. The nitrogen-rich species released from silk during the heating process can be captured by the reduced graphene shell^12^,^13^. As the result, NCMF can be obtained as a black sheet with good flexibility (Fig. S1b). In controlled experiments, pristine CMF was produced by the direct thermal treatment of silk at 600 °C. The mechanically mixed composites of CMF and thermally reduced GO with a mass ratio of 1:1 was also prepared and denoted as CMF&G.

**Figure 2:**
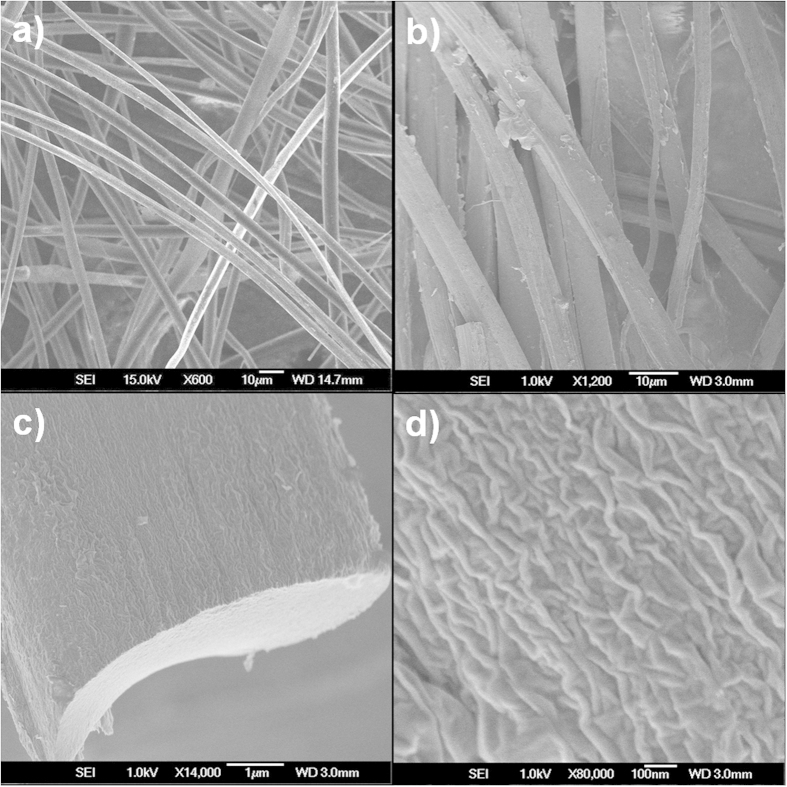
(**a**) SEM images of CMF from silk; (**b**–**d**) SEM images of NCMF at different magnifications.

The morphology and microstructure of CMF and NCMF were investigated by scanning electron microscopy (SEM) and transmission electron microscopy (TEM). The SEM images indicate that CMF has a typical fiber-like structure with a smooth surface and a uniform diameter of ~5 μm (Fig. 2a). In contrast, NCMF has a highly crumpled surface, which should be derived from the graphene shells (Fig. 2b–d). GO sheets can be viewed as 2D amphiphilic polymers with hydrophilic oxygen containing groups and hydrophobic aromatic frameworks^14^. When the oxygen containing groups of GO bind on silk via Coulombic forces, the aromatic parts of GO would tend to fold together to reduce their exposure to water and minimize the surface tension. Consequently, a wrinkled graphene shell can be formed on the surface of silk. As indicated by their SEM images, all the fibers of NCMF have the crumpled surface and no smooth parts can be observed (Fig. 2b), implying the fully enwrapping of graphene. The elemental mapping images of NCMF further disclose that N atoms are homogeneously distributed on its surface (Fig. S2). The elemental analysis results indicate that the contents of nitrogen in CMF and NCMF are calculated as 11.1 and 10.9 wt%, respectively (Table S1). The high nitrogen content of NCMF indicates that its graphene shell can capture the nitrogen containing species released from the silk during the thermal treatment and lead to an effective *in-situ* doping of nitrogen in the carbon framework, which will effectively enhance its electrochemical performance in EDLCs.

Subsequently, the X-ray diffraction (XRD) spectra of CMF and NCMF were further recorded to investigate their microstructure (Fig. S3a). Both samples exhibit obvious diffraction peaks around 26.0°, which can be indexed to the (002) diffraction plane of graphite. The much sharper diffraction peak of NCMF than that of CMF implies the higher graphitic degree of NCMF, which should be due to the addition of graphene component in NCMF during its fabrication process. The Raman spectra of CMF and NCMF show two broad peaks around 1340 and 1590 cm^−1^(Fig. S3b), which can be assigned to the D and G band of carbon, respectively^15^,^16^,^17^. The intensity ratio of D to G band (I_D_/I_G_) of NCMF is calculated to be 1.17, which is higher than that of CMF (0.98). Since the intensity of D band is related to the distortion/defects of the carbon framework^18^,^19^, the high I_D_/I_G_ ratio of NCMF should be owing to its highly wrinkled surface.

To evaluate the influence of the crumpled graphene surface on NCMF, the N_2_ sorption analysis was further carried out in this work. The N_2_ adsorption/desorption isotherms of NCMF exhibit an open loop at low relative pressures (Fig. S4a), which is probably due to the existence of very narrow slit pores in the sample^20^. It is interesting that the Brunauer-Emmett-Teller (BET) surface areas of pristine CMF and CMF&G is only 0.7 and 67 m^2^ g^−1^, respectively, while NCMF has a much enhanced BET surface area of 115 m^2^ g^−1^, which can be due to its wrinkled surface. According to the DFT model, the pore sizes of NCMF are calculated to be ranging from 1.6–4 nm (Fig. S4b).

**Figure 3:**
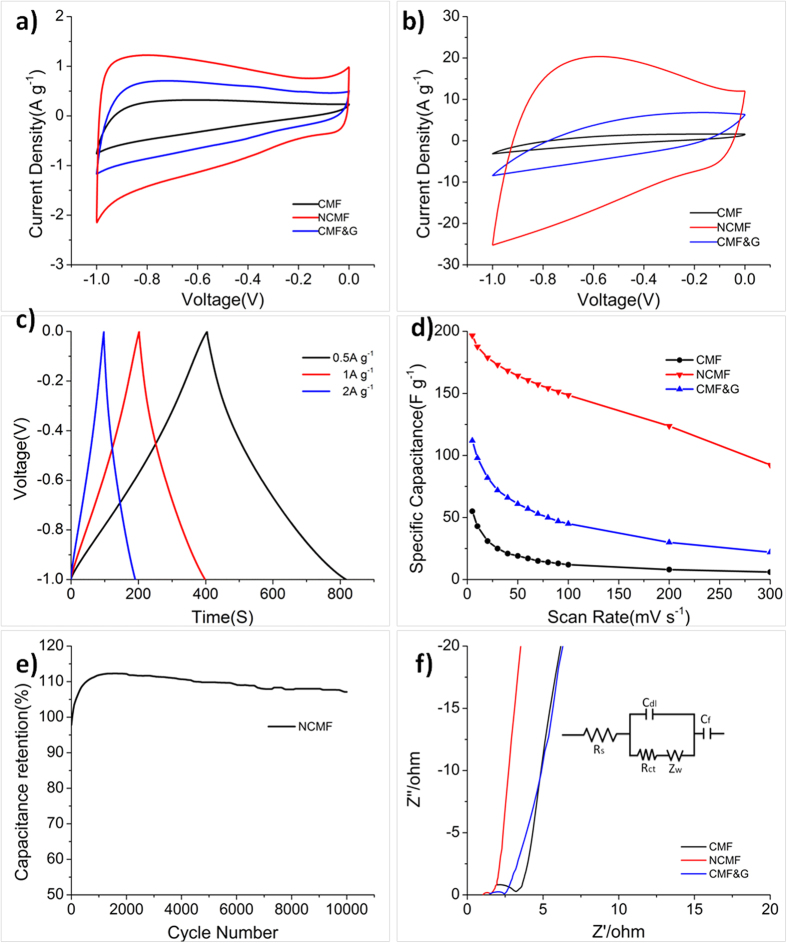
(**a**) CVs curves of CMF, NCMF and CMF&G measured at 5 mV s^−1^; (**b**) CVs curves of CMF, NCMF and CMF&G measured at 100 mV s^−1^; (**c**) galvanostatic charge-discharge curves of NCMF under different constant currents; (**d**) specific capacitances of CMF, NCMF and CMF&G at different scan rates; (**e**) cycling stability of NCMF obtained from CV curves at 300 mV s^−1^ for 10000 circles; (**f**) electrochemical impedance spectroscopy (EIS) of CMF, NCMF and CMF&G.

Given the unique features of NCMF such as highly wrinkled graphene shell and high nitrogen content, its electrochemical performance as the electrode for EDLCs was further evaluated in a three-electrode system. For comparison, CMF and CMF&G were also tested under the same conditions. Cyclic voltammetry (CV) and galvanostatic curves were first employed to probe the electrochemical behavior of the CMF, CMF&G and NCMF in 6 M aqueous KOH electrolyte at potential interval from −1 to 0 V vs. Hg/HgO electrode (Fig. 2). The CV curves of NCMF exhibit nearly symmetrical rectangular shapes and maintain well at the scan rate up to 100 mV s^**−**1^, indicative of the good double-layer capacitive behaviors (Fig. 3a,b)^21^,^22^,^23^, which can be further confirmed by their quasi triangular-shaped galvanostatic charge/discharge cycling curves (Fig. 3c). The minor distortion of the CV curves might be attributed to the existence of -COOH, -OH groups (Fig. S5). In contrast, CV profiles of CMF and CMF&G manifest highly distorted rectangular shapes at 100 mV s^**−**1^.

In order to investigate the capacitance retention of three samples at high current density, the variation of specific capacitance at different current densities is summarized. As shown in Fig. 3d, NCMF devilries a high specific capacitance as 196 F g^−1^ at 5 mV s^−1^, which is higher than those of CMF (55 F g^−1^) and CMF&G (111F g^−1^). More importantly, NCMF manifests an outstanding rate capability and its capacitance is kept as 92 F g^−1^ at 300 mV s^−1^, while those of CMF and CMF&G at the same scan rate are only 6 and 22 F g^−1^, respectively. Moreover, the cycling performance of NCMF, CMF and CMF&G were also examined from −1 to 0 V at a scan rate of 50 mV s^−1^. NCMF demonstrates an excellent electrochemical stability and keeps 94% of the initial specific capacitance after 1000 consecutive cycles (Fig. S6), outperforming CMF (81%) and CMF&G (93%). More impressively, NCMF is able to retain the initial capacitance at a high charging rate of 300 mV s^**−**1^ even after 10000 consecutive cycles (Fig. 3e).

**Figure 4:**
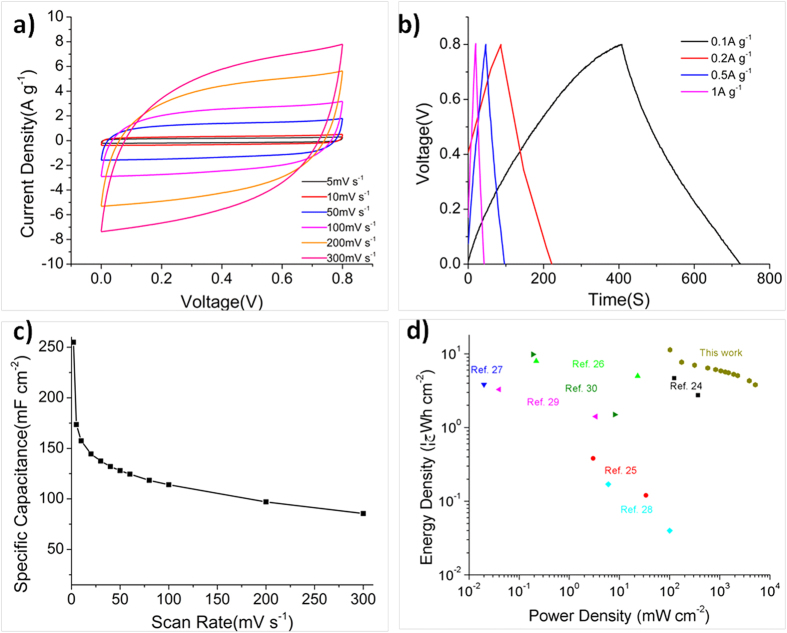
(**a**) CVs curves of the ASSS with NCMF as electrode at 5–300 mV s^−1^; (**b**) galvanostatic charge-discharge curves of the ASSS with NCMF as electrode under different constant currents; (**c**) specific capacitances of the ASSS for different scan rates; (**d**) Ragone plot of the ASSS based on two-electrode mass of active materials.

The remarkable electrochemical performance of NCMF encouraged us to further assemble all-solid-state supercapacitors (ASSS) with NCMF as electrode material and the mixed gel of polyvinyl alcohol (PVA)/H_2_SO_4_ as solid-state electrolyte. Similar to the results from the three electrode measurement, NCMF based ASSS exhibits good double-layer capacitive behaviors and its CV keeps the rectangular symmetry at different scan rates between 0 and 0.8 V, even at a scan rate as high as 300 mV s^−1^ (Fig. 4a). A high specific capacitance of 255 mF cm^−2^ is obtained at a scan rate of 2 mV s^−1^(Fig. 4b). Even at a scan rate as high as 300 mV s^−1^, its specific capacitance is still retained as 85.5 mF cm^−2^ (Fig. 4c). Calculated by integrating the CV curves at different scan rates based on the Ragone plot (Fig. 4d), the ASSS from NCMF also manifests high energy density (≈22.7 μWh cm^**−**2^ at 2 mV s^**−**1^) and power density (≈10.26 mW cm^**−**2^ at 300 mV s^**−**1^). Moreover, the exceptional electrochemical performance of the ASSS with NCMF as electrode is comparable or even higher than state of the art supercapacitors based on graphene (Fig. 4d)^24^,^25^,^26^,^27^,^28^,^29^,^30^.

These excellent electrochemical behaviors of NCMF can be attributed to the following reasons: First, the crumpled graphene shell of NCMF can provide high ionic accessible surface area for the penetration of electrolytes and thus leads to the high capacitance for the storage of charges. Moreover, the folds in the graphene shell create enormous channels and furrows on the surface of NCMF, which is favorite for the rapid movement of charge carriers along the axis of NCMF. On the other hand, the existence of the graphene shell with good mechanical stability can efficiently enhance the tolerance to the structural variation of the NCMF electrode during the charging/discharging process^31^,^32^,^33^ and thus lead to the good cycling stability of NCMF. Moreover, all of the Nyquist plots (Fig. 3f) of the three electrodes show a semicircle in the high-frequency region and straight line in the low-frequency region. The semicircle impedance plots are associated with charge transfer resistance at the electrode/solution interface, while the straight line is determined by ion diffusion. Compared with CMF and CMF&G, NCMF has the shorter radius in high frequency range and more vertical straight line lying in low frequency range, which reflects the lower resistance to mass transfer/diffusion rate of ions within the carbon framework of NCMF^34^,^35^. In addition, the nitrogen-containing functional groups of NCMF have the advantage of improving its wettability for the diffusion of the electrolyte^16^ and decreasing its internal resistance^36^. Moreover, An equivalent circuit model can be used as the equivalent circuit where R_s_ represents the ionic resistance of electrolytes, R_ct_ is the charge-transfer resistance from electrolytes moving through the electrodes during kinetically-controlled electrochemical reactions, Z_w_ is the Warburg impedance associated with diffusion of electrolytes to/from the flat electrode planes, C_dl_ is the double layer capacitance and C_f_ is the faradic capacitance (Table S2). Thus, the doped nitrogen atoms can provide a pseudo-capacitance to further improve the electrode performance of NCMF.

## Conclusion

We have successfully fabricated NCMF with highly crumpled surface via an ionic forces driven assembly of silk and GO and the following thermal treatment. The resultant NCMF possesses both a high specific surface area of 115 m^2^ g^−1^ and a high nitrogen content of 10.9 wt%. As the electrode in EDLC, NCMF shows enhanced electrochemical performance such as high specific capacitance, good rate capability, and excellent cycling stability. More importantly, our fabrication strategy provides a facile strategy to produce graphene based materials with crumpled surface for a broad range of applications in supercapacitors, secondary batteries, sensors, and catalysis.

## Methods

### Experimental Section

All the chemicals were purchased from Sinopharm Chemical Reagent Company and used as received without any further purification. Obtained from Bombyx mori cocoons, the silk fiber was purchased from Jinfeng Textile Co., Ltd, Shanghai, China. Ultrapure water (18.2 MΩ cm @25 °C) was used in all experiments.

### Synthesis of NCMF

Graphene oxide (GO) was prepared from natural graphite powders via modified Hummers method. After cut into pieces, silk was ultrasonically cleaned in acetone and water. Then the pieces of silk fiber were put into diluted HCl (0.2 M) for 1h and dried at 60 °C. Consequently, one piece of silk (200 mg) was immersed in the suspension of GO (200 mL, 1 mg mL^−1^) for 1 h. After the full combination of silk and GO, the resulting composite was dried at 40 °C for 12 h (~210 mg) and thermally treated at 600 °C for 2 h in nitrogen flow. Finally, NCMF was obtained as a black sheet.

### Characterization

Elemental analysis was measure on an elementar (Vario Micro cube, Germany). Transmission electron microscopy (TEM) measurements were conducted on a JEM-2010F (JEOL, Japan) operated at 200 kV. Samples were suspended in ethanol and transferred onto a Cu grid for TEM measurements. Field emission-scanning electron microscopy (FE-SEM) images were taken on a JSM-7401F (JEOL Ltd., Japan) microscope. Nitrogen sorption isotherms were measured at 77 K with an Autosorb-1MP instrument (Quantachrome, USA).

## Additional Information

**How to cite this article**: Liu, R. *et al*. Nitrogen-doped Carbon Microfiber with Wrinkled Surface for High Performance Supercapacitors. *Sci. Rep.* **6**, 21750; doi: 10.1038/srep21750 (2016).

## Supplementary Material

Supplementary Information
