# Supplementary material for: Nitrogen-doped Carbon Microfiber with Wrinkled Surface for High Performance Supercapacitors
Source: Sci Rep. 2016 Feb 18;6:21750. doi: 10.1038/srep21750 (PMC4757873; doi:10.1038/srep21750)
Supplement: Supplementary Information [file srep21750-s1.pdf]

**Supporting information**

# Nitrogen-doped Carbon Microfiber with Wrinkled Surface for High Performance Supercapacitors

Ruili Liu*[1,2], Lixia Pan[2], Jianzhong Jiang[3], Xin Xi[2], Xiaoxue Liu[2], and Dongqing Wu*[3]

[1] *State Key Laboratory of Advanced Optical Communication Systems and Networks, Department of Electronic Engineering, Shanghai Jiao Tong University, Shanghai 200240, China. E-mail: ruililiu@sjtu.edu.cn*

[2] *Department of Chemical Engineering, School of Environment and Chemical Engineering, Shanghai University, Shanghai 200444, China.*

[3] *School of Chemistry and Chemical Engineering, Shanghai Jiao Tong University, Shanghai 200240, China. E-mail: wudongqing@sjtu.edu.cn*

***Electrochemical Measurement:***

All the electrochemical experiments were carried out using CHI 660e workstation (Chenhua, Shanghai). In a three-electrode system, 6 M KOH was used as the aqueous electrolyte, a platinum plate as the counter electrode, and a Hg/HgO electrode as the reference electrode. To prepare the working electrode, the active materials were pressed (15 MPa) between two pieces of nickel foam with area of 1 $cm^2$. In the case of all-solid-state supercapacitors (ASSSs), gel-like electrolyte was first fabricated by mixing $H_2SO_4$ (6 g) and PVA (6 g) in deionized water (60 mL) and thus heated up to 80 °C under vigorous stirring for about 3 hours until the solution became clear. Two slices of platinum plate (1*1 $cm^2$) were painted with the active material (thickness: about 40 μm) on each plate, and then immersed in the PVA/$H_2SO_4$ electrolyte. The resulting electrolyte-filled electrodes were solidified overnight at room temperature. Finally, as-prepared two electrodes were symmetrically face to face integrated into one ASSS.

The ASSS of NCMF were examined by cyclic voltammetry (CV) and galvanostatic charge-discharge. The capacitance of electrode was calculated from the voltammetric responses, according to the following equation (1):

$$C = \frac{\int I\mathrm{d}V}{2mS\Delta U} \qquad (1)$$

where *C* is denoted as the capacitance contribution from NCMF electrode, *I* is the voltammetric discharge current (in amperes), $S$ is the scan rate (in V $s^{-1}$), ΔU is the absolute value of potential window (in V), and $m$ is the total mass of the active material (g).

The electrochemical performance of ASSS shown in the Ragone plot was based on the capacitance from equation (1). The energy density of the device was obtained from the formula given in equation (2):

$$E = \frac{1}{2} \times C \times \frac{(\Delta V))^2}{3,600} \times \frac{m}{s} \qquad (2)$$

where *E* is the energy density (in Wh $cm^{-2}$), *C* is the capacitance obtained from equation (1) , $\Delta V$ is the discharge voltage range (in volts), s is the electrode area (in $cm^2$) and $m$ is the total mass of the active material (g).

The powder density was calculated from the formula given in equation (3):

$$P = \frac{E}{\Delta t} \times 3600 \qquad (3)$$

where *P* is the powder density (in W $cm^{-2}$), E is the energy density obtained from equation (3) and $\Delta t$ is the discharge time (s).

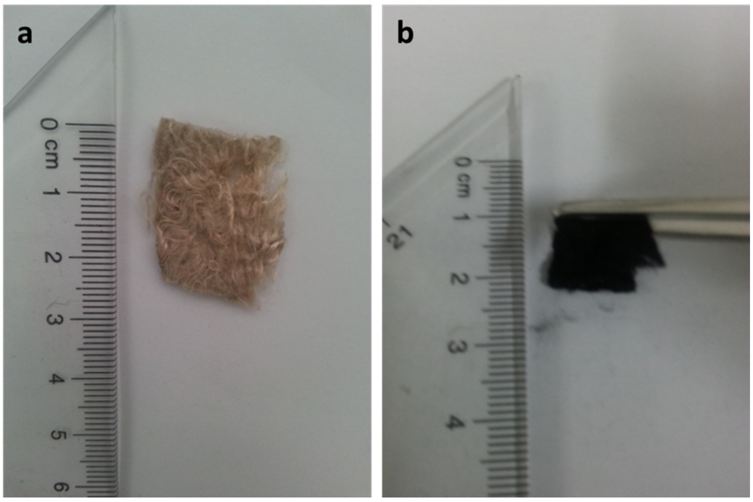


**Figure S1.** (a) digital photograph of the core/shell structured silk/GO composite and (b) NCMF.

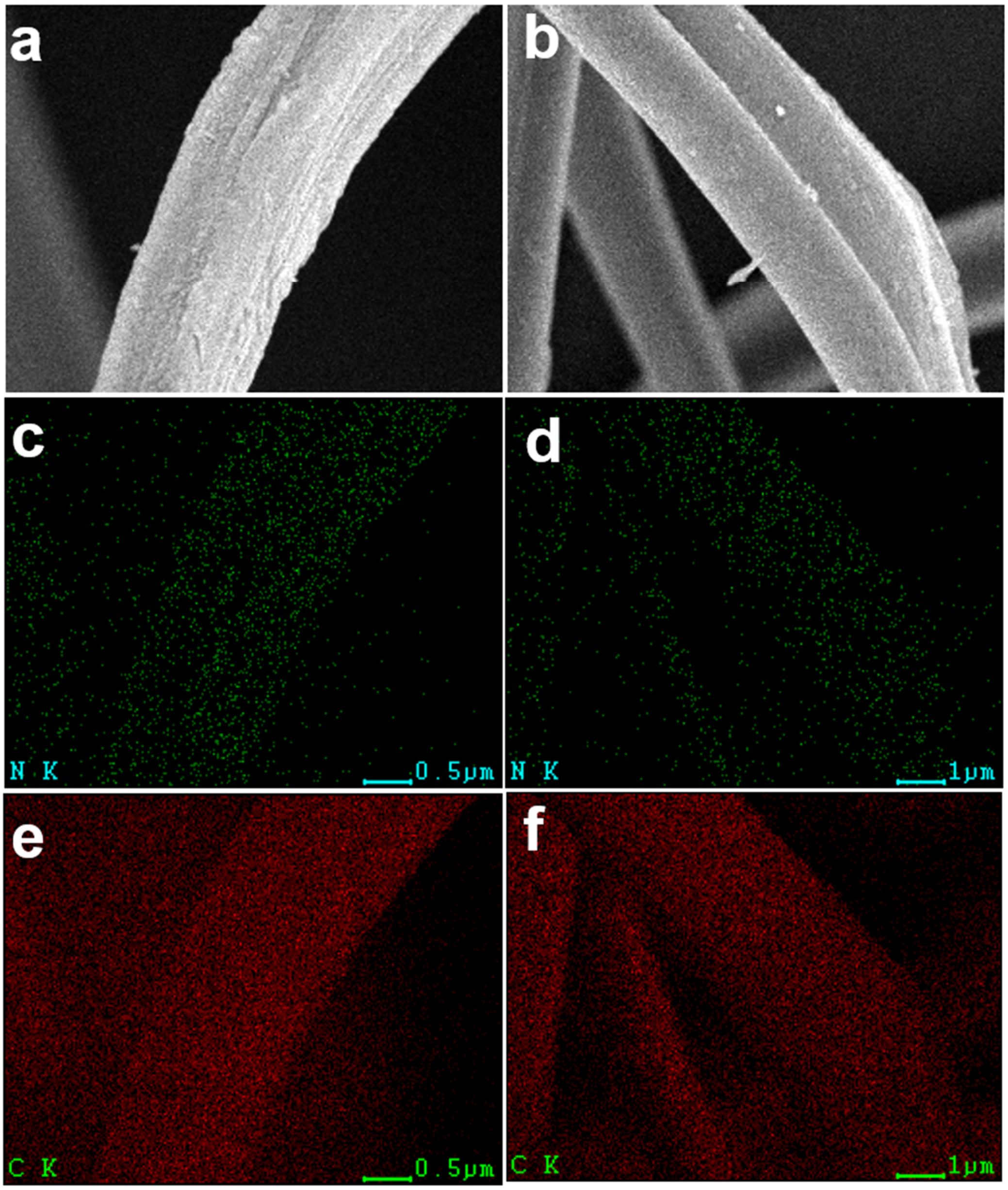


**Figure S2.** (a) and (b) SEM images of NCMF, (c), (d), (e) and (f) elemental mapping images of NCMF.

**Table S1**. Surface areas and compositions of CMF and NCMF

| **Sample** | **Surface area** $m^2/g$ | **N** wt% | **C** wt% | **H** wt% | **Capacitance** F/g at 50 mV $s^{-1}$ | **Durability** after 1000 cycles % |
|---|---|---|---|---|---|---|
| **CMF** | 0.7 | 11.11 | 73.99 | 2.22 | 55 | 81 |
| **NCMF** | 115 | 10.94 | 63.33 | 2.85 | 196 | 94 |

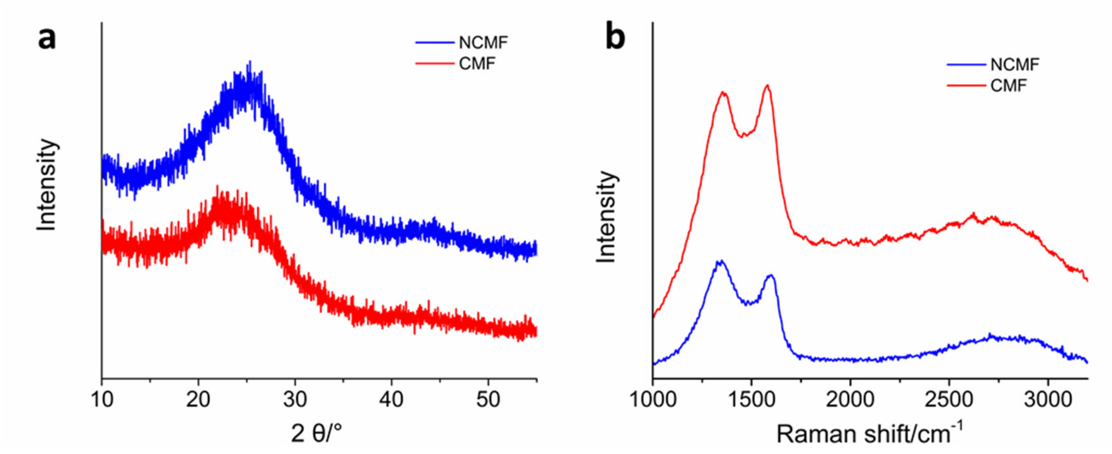


**Figure S3.** a) XRD spectra of NCMF and CMF; b) Raman spectra of NCMF and CMF.

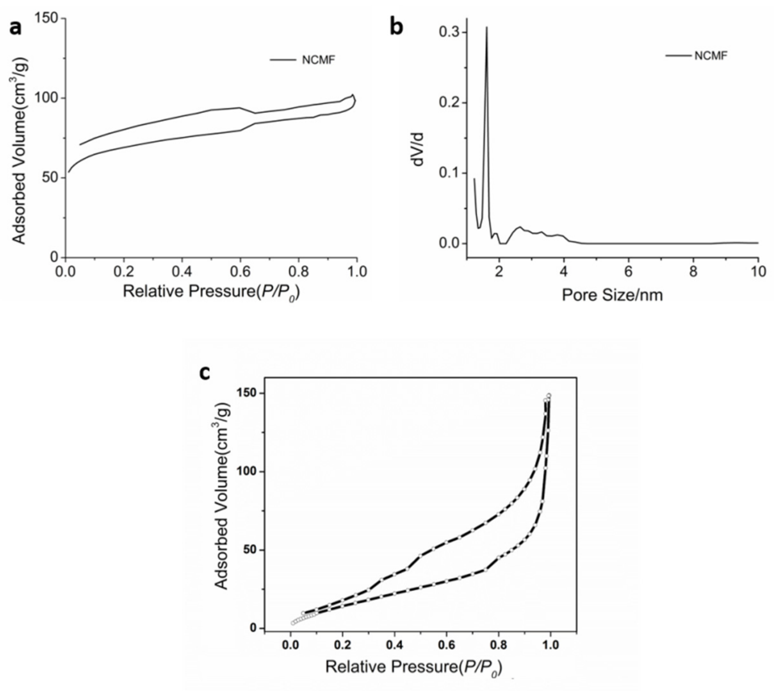


**Figure S4.** a) $N_2$ adsorption-desorption isotherm and b) the pore size distribution of NCMF calculated by DFT method; c) $N_2$ adsorption-desorption isotherm of CMF&G

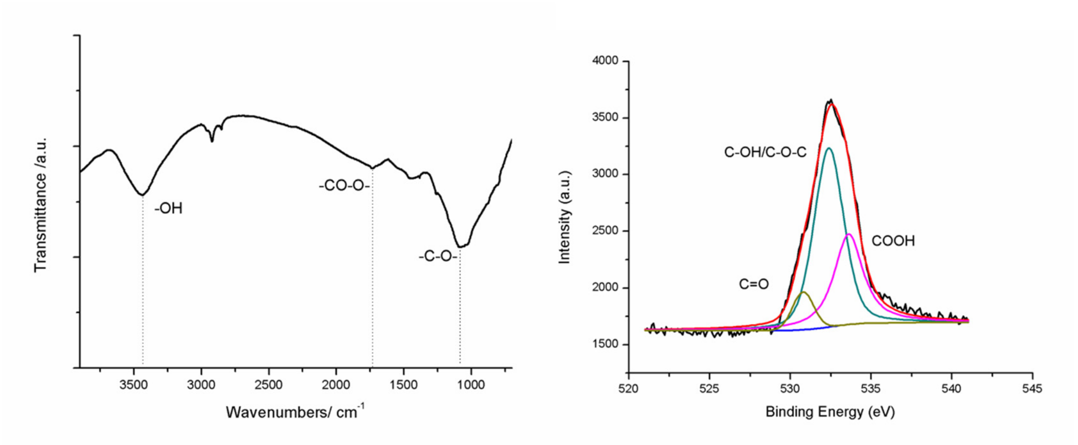


**Figure S5.** (a) FTIR and (b) O 1s XPS spectra of NCMF

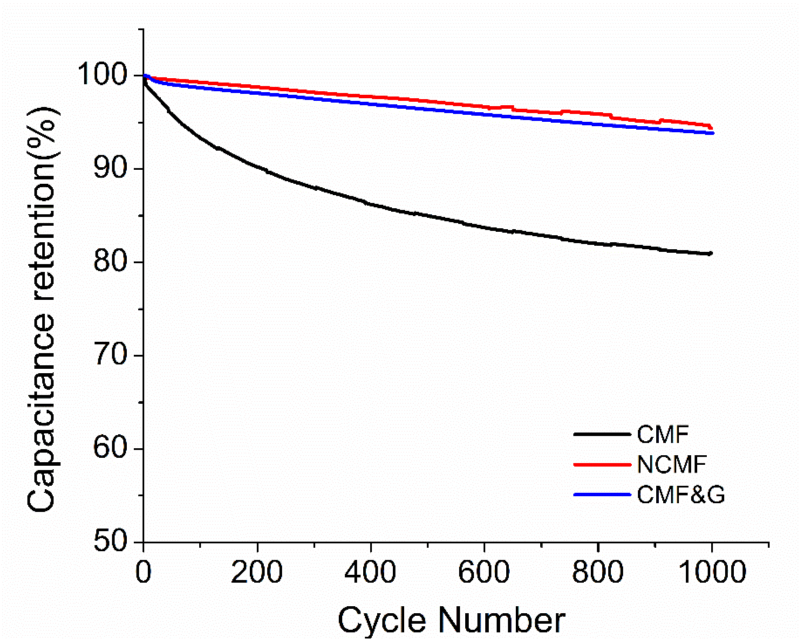


**Figure S6.** Cycling performance of NCMF, CMF and CMF&G at 50 mV $s^{-1}$

**Table S2** The component of the equivalent circuit fitted for the impedance spectra.

| Sample | Rs(Ω $cm^2$) | Rct(Ω $cm^2$) | Zw(Ω $s^{-0.5}cm^2$) |
|---|---|---|---|
| CMF | 1.52 | 162.2 | 21.5 |
| NCMF | 1.07 | 4.29 | 0.85 |
| CMF&G | 1.61 | 0.71 | 0.91 |
